# Fatal infection in three Grey Slender Lorises (*Loris lydekkerianus nordicus*) caused by clonally related *Trueperella pyogenes*

**DOI:** 10.1186/s12917-017-1171-8

**Published:** 2017-08-29

**Authors:** Samy Nagib, Stefanie P. Glaeser, Tobias Eisenberg, Osama Sammra, Christoph Lämmler, Peter Kämpfer, Nicole Schauerte, Christina Geiger, Ute Kaim, Ellen Prenger-Berninghoff, André Becker, Amir Abdulmawjood

**Affiliations:** 1 Institut für Pharmakologie und Toxikologie, Justus-Liebig-Universität Gießen, Schubertstraße 81, D-35392 Gießen, Germany; 2 Institut für Angewandte Mikrobiologie, Justus-Liebig-Universität Gießen, Heinrich-Buff-Ring 26-32, D-35392 Gießen, Germany; 3 Landesbetrieb Hessisches Landeslabor, Schubertstraße. 60, D-35392 Gießen, Germany; 4 Zoologischer Garten Frankfurt am Main, Bernhard-Grzimek-Allee 1, D-60316 Frankfurt, Germany; 5 Institut für Hygiene und Infektionskrankheiten der Tiere, Justus-Liebig-Universität Gießen, Frankfurter Straße. 85-91, D-35392 Gießen, Germany; 6 Institute of Food Quality and Food Safety, Research Center for Emerging Infections and Zoonoses (RIZ), University of Veterinary Medicine Hannover, Foundation, Bischofsholer Damm 15, D-30173 Hannover, Germany

**Keywords:** *Trueperella pyogenes*, grey slender loris, *Loris lydekkerianus nordicus*, lorises, virulence genes, clonal relationship, DNA fingerprint, multilocus sequence analysis

## Abstract

**Background:**

*Trueperella pyogenes* is a worldwide known bacterium causing mastitis, abortion and various other pyogenic infections in domestic animals like ruminants and pigs. In this study we represent the first case report of three unusual fatal infections of Grey Slender Lorises caused by *Trueperella pyogenes*. Meanwhile, this study represents the first in-depth description of the multilocus sequence analysis (MLSA) on *T. pyogenes* species*.*

**Case presentation:**

Three *Trueperella pyogenes* were isolated from three different Grey Slender Lorises, which died within a period of two years at Frankfurt Zoo (Frankfurt am Main - Germany). The three Grey Slender Loris cases were suffering from severe sepsis and died from its complication. During the bacteriological investigation of the three cases, the *T. pyogenes* were isolated from different organisms in each case. The epidemiological relationship between the three isolates could be shown by four genomic DNA fingerprint methods (ERIC-PCR, BOX-PCR, (GTG)_5_-PCR, and RAPD-PCR) and by multilocus sequence analysis (MLSA) investigating four different housekeeping genes (*fusA-tuf-metG-gyrA).*

**Conclusion:**

In this study, we clearly showed by means of using three different rep-PCRs, by RAPD-PCR and by MLSA that the genomic fingerprinting of the investigated three *T. pyogenes* have the same clonal origin and are genetically identical. These results suggest that the same isolate contaminated the animal’s facility and subsequently caused cross infection between the three different Grey Slender Lorises. To the best of our knowledge, this is the first epidemiological approach concentrating on *T. pyogenes* using MLSA.

## Background

*Trueperella pyogenes* is a well-known pathogen of domestic ruminants and pigs causing mastitis, abortion and a variety of pyogenic infections [1]. As summarised by Jost and Billington [2] this bacterial pathogen is also able to cause diseases in a large number of different animal species including antelopes, bisons, camels, chickens, deer, elephants, gazelles, horses, macaws, reindeer, turkeys and wildebeest and also in companion animals such as dogs and cats. In 2010, Ülbegi-Mohyla et al. [3] characterised two *T. pyogenes* isolated from septicaemia of a gecko and a bearded dragon both phenotypically and genotypically. In 2012, Oikonomou et al. [4] reported that *T. pyogenes* is one of mainly frequently isolated pathogens from the investigated mastitis cases. The *T. pyogenes* acts as an opportunistic pathogen, causing endometritis in dairy cattle once the protective epithelium has been lost after parturition [5]. In later research, the complete genome sequencing of *T. pyogenes* was undertaken from a field isolate of a dairy cow suffering from metritis [6, 7] and from infected goats [8]. Grey Slender Lorises (*Loris lydekkerianus nordicus*) are a primate species from the family *Lorisideae* whose taxonomy is currently under revision. Their habitat is East and South India as well as Sri Lanka [9]. Grey Slender Lorises are primarily insectivorous nocturnal animals with loose social interactions. They forage on trees in dry zone forests where they also sleep in aggregations of several animals. In 2012, a *T. pyogenes* 11-07-D-03394 was isolated from a facial abscess of a six-year-old Grey Slender Loris kept in a terrarium at Frankfurt Zoo [10].

## Case presentation

The Grey Slender Lorises in the present study originated from a European Association of Zoo and Aquaria (EAZA) breeding programme and were living at Frankfurt Zoo. The Lorises were kept in pairs in the nocturnal animal house. In October 2011, *T. pyogenes* 11-07-D-03394 was isolated, as previously described [10], from a facial abscess of a six-year-old male Grey Slender Loris. In May 2012, a second *T. pyogenes* 121,008,157 was isolated from a nasal swab of a ten-year-old male Grey Slender Loris which was living in the same terrarium and was suffering from purulent rhinitis. The *T. pyogenes* 121,008,157 was isolated together with *Enterococcus* spp*.*, *Pasteurella* spp*.*, *Pseudomonas aeruginosa* and coliform bacteria. After two weeks, this Grey Slender Loris died and *T. pyogenes* was isolated postmortem together with *Enterococcus faecalis, Klebsiella pneumoniae* and *Escherichia coli* from the skull of this animal. *E. faecalis, K. pneumoniae* and *E. coli* were additionally isolated from the liver, kidney, lung, spleen, tongue, brain and nasal mucosa. The post mortem analysis of the animal revealed a catarrhal purulent rhinitis, a catarrhal purulent sinusitis associated with an extended purulent catarrhal pneumonia, an acute fibrinous-purulent pericarditis as well as an interstitial nephritis. The animal had also lost two upper left molars and three top right molars. Later on, in December 2012, a third Grey Slender Loris (9.5 year old female) died in the same terrarium also from bacterial septicaemia following a dental abscess. In postmortem microbiological examinations, the *T. pyogenes* was isolated together with *E. faecalis* from the liver, kidney, lung, intestine, vagina and orbita and in high numbers from paranasal sinuses and dental cavities. The *T. pyogenes* isolate 121,018,522 used for further studies was isolated from the paranasal sinuses of this animal. Post mortem analysis of the animal also revealed a chronic nephritis of both kidneys. The animal had similar teeth problems as the two previously mentioned lorises. The bacteriological carcass inspection of the deceased lorises found *T. pyogenes* to be the predominating bacteria. In the present study the first isolate *T. pyogenes* 11-07-D-03394 obtained from the first case and the two *T. pyogenes* isolates (121,008,157 and 121,008,522) obtained from the second and third cases were identified both phenotypically and genotypically. Additionally, all three isolates were investigated for epidemiological relationships by means of various genotypical tests. For control purposes the strains *T. pyogenes* DSM 20630^T^, *T. pyogenes* DSM 20594 and *Arcanobacterium haemolyticum* DSM 20595^T^ were included.

### Phenotypic and genotypic identification

Both newly isolated *T. pyogenes* were identified phenotypically as described by Eisenberg et al. [10]. A genotypic identification of all three *T*. *pyogenes* isolates was subsequently performed by sequencing the 16S rRNA gene [10–13] and the glyceraldehyde-3-phosphate dehydrogenase encoding gene *gap* [13]. Furthermore, the three *T*. *pyogenes* isolates were genotypically identified by amplifying the *T*. *pyogenes* species-specific region of the 16S-23S rRNA intergenic spacer region (ISR) and the *T*. *pyogenes* species-specific region of the superoxide dismutase A encoding gene *sodA*. The three *T*. *pyogenes* isolates were additionally characterised by amplifying of several known and putative virulence factor encoding genes. These virulence genes included gene *plo* encoding pyolysin, gene *cbpA* encoding a collagen-binding protein, gene *nanH* encoding neuraminidase H, gene *nanP* encoding neuraminidase P and the fimbriae encoding genes *fimA*, *fimC* and *fi*mE [3, 11, 12]. In addition, tetracycline resistance encoding gene *tet(W)* was amplified as described by Zastempowska and Lassa [14].

### Genomic fingerprinting

The genomic fingerprinting of the three *T*. *pyogenes* isolates originating from the lorises was performed using four genomic DNA fingerprinting methods. These included three repetitive element primed (rep)-PCRs [ERIC-PCR, BOX-PCR and (GTG)_5_-PCR] and random amplification polymorphic DNA (RAPD-PCR) analysis as described in detail by Glaeser et al. [15]. The sequences of the oligonucleotide primers and the thermocycler programs are summarised in Table 1. Cluster analysis of genomic fingerprinting patterns was performed in GelCompar II version 4.5 (Applied Maths) using the unweighted pair-group method using the arithmetic average (UPGMA) clustering method based on the Pearson correlation (0.5% optimisation; 1% position tolerance), which considers the presence/absence and the intensity of DNA bands. A consensus matrix was calculated and a composite clustering was performed.

**Table 1:** The Oligonucleotide primer sequences and PCR conditions used in the present study

Oligonucleotide primers	Sequence	Program^a^
1. 16S rDNA UNI-L	5′-AGA GTT TGA TCA TGG CTC AG-3′	1
2. 16S rDNA UNI-R (Amplification primer)	5′-GTG TGA CGG GCG GTG TGT AC-3′	
3. 16S rDNA-533F	5′-GTG CCA GCM GCC GCG GTA A-3′	–
4. 16S rDNA-907R (Sequencing primer)	5′-CCG TCA ATT CMT TTG AGT TT-3′	
5. Gap-F	5′-TCG AAG TTG TTG CAG TTA ACG A-3′	2
6. Gap-R	5′-CCA TTC GTT GTC GTA CCA AG-3′	
7. ERIC1RF	5′-ATG TAA GCT CCT GGG GAT TCA C-3′	3
8. ERIC2	5′-AAG TAA GTG ACT GGG GTG AGC-3′	
9. BOXA1R	5′-CTA CGG CAA GGC GAC GCT GAC G-3′	3
10. (GTG)_5_	5′-GTG GTG GTG GTG GTG-3′	4
12. RAPD primer B	5′- ATC TGG CAG C − 3′	5
13. fusA-F	5′-GCT TCA TCA ACA AGA TGG AC-3′	6
14. fusA-R	5′-CTC GAT TG CGA CGT GG AT-3′	
15. tuf-F	5′-GGA CGG TGA TTG GAG AAG AAT GG-3′	7
16. tuf-R	5′-CCA GGT TGA TTA CGC TCC AGA AGA-3′	
17. metG-F	5′-GCC GGT TTT GGT GTT CC-3′	8
18. metG-R	5′-GGC CAA ATC TGG GAA TGG-3′	
19. gyrA-F	5′-CCA CCA GAT CGA GGT CAT C-3′	9
20. gyrA-R	5′-TCG TCG GCA GTG AAA CGC A-3′	

^a^PCR Program 1: ×1 (95 °C, 600 s), ×30 (95 °C, 30 s, 58 °C, 60 s, 72 °C, 60 s), ×1 (72 °C, 420 s). 2: ×1 (94 °C, 180 s), ×30 (94 °C, 30 s, 50 °C, 40 s, 72 °C, 60 s), ×1 (72 °C, 300 s). 3: ×1 (95 °C, 180 s), ×30 (94 °C, 30 s, 53 °C, 60 s, 70 °C, 480 s), ×1 (72 °C, 960 s). 4: ×1 (95 °C, 180 s), ×30 (94 °C, 30 s, 53 °C, 60 s, 70 °C, 180 s), ×1 (72 °C, 960 s). 5: ×1 (95 °C, 180 s), ×45 (94 °C, 15 s, 34 °C, 60 s, 70 °C, 120 s), ×1 (72 °C, 600 s). 6: ×1 (94 °C, 180 s), ×30 (94 °C, 45 s, 57 °C, 30 s, 72 °C, 90 s), ×1 (72 °C, 420 s). 7: ×1 (94 °C, 180 s), ×30 (94 °C, 45 s, 57 °C, 40 s, 72 °C, 60 s), ×1 (72 °C, 420 s). 8: ×1 (94 °C, 180 s), ×30 (94 °C, 45 s, 52 °C, 30 s, 72 °C, 90 s), ×1 (72 °C, 600 s). 9: ×1 (94 °C, 180 s), ×30 (94 °C, 45 s, 52 °C, 30 s, 72 °C, 90 s), ×1 (72 °C, 600 s)

### Multilocus sequence analysis (MLSA)

This represents the first MLSA-based study applied to field isolates of the species *T. pyogenes*. The MLSA was performed with the target genes translation elongation factor G encoding gene *fusA,* translation elongation factor Tu encoding gene *tuf*, methionyl-tRNA synthetase encoding gene *metG* and DNA gyrase, subunit A encoding gene *gyrA.* The sequences of the oligonucleotide primers for amplifying the four housekeeping genes were designed with the sequence data of the *A. haemolyticum* DSM 20595^T^ genome project [16]. The target genes, the sequences of the oligonucleotide primers and the thermocycler programs are summarised in Table 1. Sequences of partial genes were concatenated in the following order: *fusA* (746 nt), *tuf* (795 nt), *metG* (836 nt) and *gyrA* (937 nt). Analyses were performed at the nucleotide and amino acid sequence level with *T. pyogenes* DSM 20630^T^, *T. pyogenes* DSM 20594 and *A. haemolyticum* DSM 20595^T^ as controls. MLSA analysis was performed using the MEGA version 6 [17]. Full-length gene sequences from the genome of *A. haemolyticum* DSM 20595^T^ (CP002045) were used as reference sequences to obtain the correct open reading frame (ORF) for translation into amino acid sequences. Alignments of nucleotide and the amino acid sequences were performed with MUSCLE implemented in MEGA version 6 [18]. The phylogenetic trees in single gene base analysis of the four target genes, respectively, were constructed with the neighbour-joining method [19] using the Kimura-2-parameter model for nucleotide sequences [20] or the JTT matrix-based method for amino acid sequences [21]. The phylogenetic trees of concatenated sequences were constructed using the maximum-likelihood method based on evolutionary distances calculated with the general time reversible model for nucleotide sequences [22] and again with the JTT matrix-based method for amino acid sequences. A discrete gamma distribution was used to model evolutionary rate differences among sites (5 categories; +G) and a rate variation model allowed for some sites to be evolutionarily invariable (+I). Nucleotide and amino acid sequence similarities of single and concatenated genes were determined based on p-distances calculated in MEGA version 6.

## Results and discussion

**Fig. 1:**
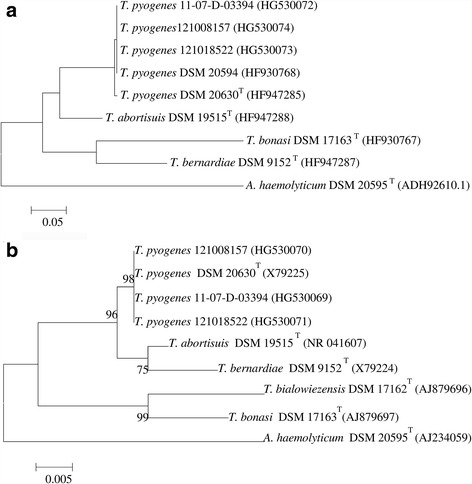
Phylogenetic trees based on sequences of 16S rRNA (**a**) and glyceraldehyde-3-phosphate dehydrogenase encoding gene *gap* (**b**) of the three *T. pyogenes* strains of the present study and reference strains of the genra *Trueperella* and *Arcanobacterium* obtained from GenBank (NCBI). Trees were constructed using the maximum-likelihood method based on evolutionary distances calculated with the general time reversible model. Numbers at branch nodes represent the percentage of replicate trees in which the associated taxa clustered together in bootstrap tests (1000 replicates). Only bootstrap values ≥70% are shown

Compared to previously identified *T. pyogenes* [23], the already described *T. pyogenes* 11-07-D-03394 [10] and the newly investigated *T. pyogenes* 121,008,157 and *T. pyogenes* 121,018,522 in the present study, could be identified by determining hemolysis and CAMP-like haemolytic reactions, by various other phenotypical and genotypical tests, together with *T. pyogenes* 11-07-D-03394, by sequencing 16S rRNA and *gap* genes (Fig. 1), and by amplifying of *T*. *pyogenes* species-specific regions of ISR and superoxide dismutase A encoding gene *sodA* as molecular targets, respectively (Table 2). The three loris isolates and the type strain *T. pyogenes* DSM 20630^T^ shared 100% 16S rRNA gene sequence similarity and 95% to 99% 16S rRNA gene sequence similarity with type strains of other species of the genera *Trueperella* and *Arcanobacterium* (98.8% similarity with *T. abortisuis* DSM 19515^T^, 94.6% similarity with *A. haemolyticum* DSM 20595^T^). In addition, as shown in Fig. 1, the three loris isolates and *T. pyogenes* DSM 20594 shared identical *gap* gene sequences, with 99% sequence similarity to *T. pyogenes* DSM 20630^T^ and 70% to 88% sequence similarity to the type strains of other species of the genera *Trueperella* and *Arcanobacterium* (88% similarity to *T. abortisuis* DSM 19515^T^, 73% similarity to *A. haemolyticum* DSM 20595^T^).

**Table 2:** Phenotypical and genotypical properties of the investigated *T. pyogenes*

	*T. pyogenes* 121,018,522	*T. pyogenes* 121,008,157	*T. pyogenes* 11-07-D 03394	*T. pyogenes* DSM 20630^T^*	*T. pyogenes* DSM 20594*
Phenotypical properties					
Hemolysis on sheep blood agar	+	+	+	+	+
Hemolysis on rabbit blood agar	+	+	+	+	+
CAMP-like reaction with:					
*Staphylococcus aureus* β-hemolysin	+	+	+	+	+
*Streptococcus agalactiae*	−	−	−	−	−
*Rhodococcus equi*	+	+	+	+	+
Reverse CAMP reaction	−	−	−	−	−
Nitrate reduction^1^	−	−	−	−	−
Pyrazinamidase^1^	−	−	−	−	−
Pyrrolidonyl arylamidase^1^	+	+	+	+	+
Alkaline phosphatase^1^	+	+	+	−	−
β-Glucuronidase (β-GUR)^1,2,3^	+	+	+	+	+
α-Galactosidase (α-GAL)^2^	−	−	−	−	−
β-Galactosidase (β-GAL)^1,3^	+	+	+	+	+
α-Glucosidase (α-GLU)^1,2,3^	+	+	+	+	+
β-Glucosidase (β-GLU)	−	−	−	−	−
N-acetyl- β-Glucosaminidase (β-NAG)^1,3^	+	+	+	+	+
Esculin (β-Glucosidase)^1^	−	−	−	−	−
Urease^1^	−	−	−	−	−
Gelatine^1,4^	−	−	−	+	+
Fermentation of:					
D-Glucose^1^	+	+	+	+	+
D-Ribose^1^	+	+	+	+	+
D-Xylose^1^	+	+	+	+	+
D-Mannitol^1^	−	−	−	−	−
D-Maltose^1^	+	+	+	+	+
D-Lactose^1^	+	+	+	+	+
Glycogen^1^	+	+	+	+	−
α-Mannosidase^2^	−	−	−	−	−
Catalase	−	−	−	−	−
Serolysis on Loeffler agar	+	+	+	+	+
Caseinase	+	+	+	+	+
Starch hydrolysis (amylase)	+	+	+	+	−
Cross reaction with streptococcal serogroup G specific antiserum	+	+	+	+	+
Genotypical properties					
*T. pyogenes* 16S rRNA gene sequence	+	+	+	+	+
*T. pyogenes* gene gap sequence	+	+	+	+	+
*T. pyogenes* specific part of ISR	+	+	+	+	+
*T. pyogenes* specific part of gene *sodA*	+	+	+	+	+
Pyolysin encoding gene *plo*	+	+	+	+	+
Collagen-binding protein encoding gene *cbpA*	−	−	−	+	−
Neuraminidase H encoding gene *nanH*	+	+	+	+	+
Neuraminidase P encoding gene *nanP*	−	−	−	+	+
Fimbriae endoding gene *fimA*	+	+	+	−	+
Fimbriae endoding gene *fimC*	+	+	+	+	+
Fimbriae endoding gene *fimE*	+	+	+	+	+
Tetracycline resistance encoding gene *tet(W)*	+	+	+	+**	−**

The reactions are shown as follows:* = synergistic CAMP-like reaction with staphylococcal β-hemolysin and *Rhodococcus equi* as indicator strains; ** = results mostly obtained from Eisenberg et al. [19]; +, positive reaction; −, negative reaction. 1 = Api-Coryne test system (Biomerieux, Nürtingen, Germany); 2 = tablets containing substrates (Rosco Diagnostica A/S, Taastrup, Denmark); 3 = 4-methylumbelliferyl conjugated substrates (Sigma, Steinheim, Germany)

To demonstrate the clonal lineage and the epidemiological relationship, the *T. pyogenes* isolates from this study were further found to possess known and putative virulence factor encoding genes *plo*, *nanH*, *fimA*, *fimC*, *fimE* and *tet*(*W*)*.* The genes *cbpA* and *nanP* could not be detected by the amplification with the applied primer systems (Table 2). The possible importance of the known and putative virulence factors has been discussed previously [2, 10, 11].

**Fig. 2:**
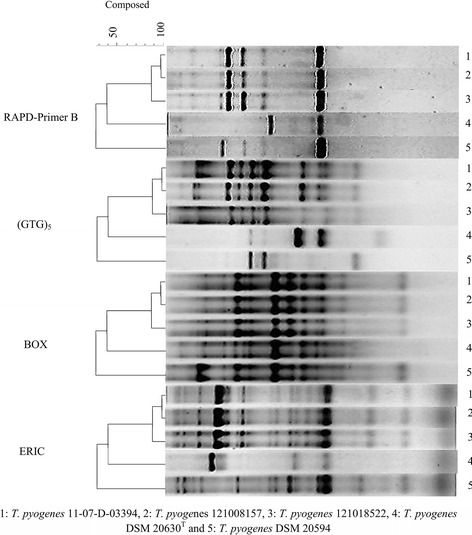
Genomic fingerprint pattern of the three Grey Slender Lorises strains in comparison to *T. pyogenes* reference strains with three different (REP)-PCRs (ERIC-PCR, BOX-PCR, and (GTG)^5^–PCR) and random amplification polymorphic DNA (RAPD-PCR)

The *T*. *pyogenes* were also investigated for an epidemiological relationship by four genomic fingerprinting methods, three (rep)-PCRs and one RAPD-PCR, respectively. (rep)-PCRs were introduced to differentiate microbes by combining the advantage of DNA amplification with the application of repetitive sequence based oligonucleotide primers. RAPD fingerprinting has also been described as a useful tool for analysing the genetic structure of closely related bacteria. Since RAPD fingerprints target the whole genome, this method is far more sensitive in terms of detecting genetic diversity than, for example, 16S rRNA gene sequencing [24]. The genomic fingerprint analysis undertaken in the present study yielded for all three *T*. *pyogenes* isolates of loris origin uniform patterns in ERIC-PCR, BOX-PCR, (GTG)_5_-PCR, and in RAPD-PCR analysis. The patterns differed from those of *T. pyogenes* DSM 20630^T^ and *T. pyogenes* DSM 20594 (Fig. 2) indicating that the three isolates are genetically identical and therefore of same clonal origin, while the other strains are somehow genetically different. All four techniques had already been used successfully for molecular typing of various bacterial species [25–31]. BOX-PCR typing was used for DNA fingerprinting of a variety of bacteria including *T. pyogenes* from musk deer [32].

**Fig. 3:**
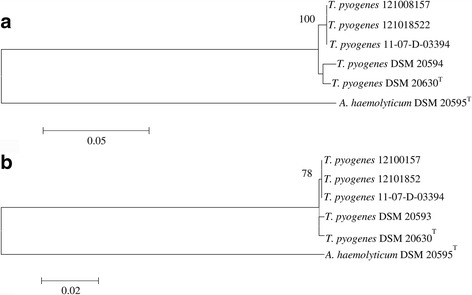
Phylogenetic analysis based on concatenated partial *fusA-tuf-metG-gyrA* nucleotide sequences of a total of 3314 nucleotide positions (**a**) and FusA-Tuf-MetG-GyrA amino acid sequences of a total of 1103 amino acid positions (**b**) of the three investigated target genes of the three *T. pyogenes* of Grey Slender Loris origin, *T. pyogenes* DSM 20594, *T. pyogenes* DSM 20630^T^ and *A. haemolyticum* DSM 20595^T^. Trees were constructed using the maximum-likelihood method based on evolutionary distances calculated with the general time reversible model (for nucleotide sequences) or the JTT matrix-based method (for amino acid sequences). Numbers at branch nodes represent the percentage of replicate trees in which the associated taxa clustered together in bootstrap tests (1000 replicates). Only bootstrap values ≥70% are shown

The clonal relationship of the three *T*. *pyogenes* of loris origin could also be demonstrated by MLSA, investigating the target genes *fusA, tuf, metG* and *gyrA.* All four partially sequenced nucleotide sequences and amino acid sequences of the housekeeping genes were concatenated in the following order: *fusA-tuf-metG*-*gyrA* and FusA-Tuf-MetG-GyrA with a nucleotide sequence of 3314 bp and an amino acid sequence of 1103 aa. The targeted amplified DNA fragments were double sequenced on both strands and the sequences were deposited in the GenBank (National Center for Biotechnology Information) (Table 3). The phylogenetic analysis was based on the combined utilisation of the *fusA-tuf-metG-gyrA* partial gene sequences. The GenBank accession numbers of locus sequences obtained in this study are provided in Table 3. The cluster analyses of the phylogenetic trees of concatenated sequences succeeded in subdividing the three investigated *T*. *pyogenes* isolates originating from the three Grey Slender Lorises and the reference strains (*T. pyogenes* DSM 20594, *T. pyogenes* DSM 20630^T^ and *A. haemolyticum* DSM 20595^T^). The concatenated sequence of the four loci on the nucleotide level among the 6 investigated isolates and strains with the corresponding 3314 bp resulted in 2468 conserved, 846 variable and 28 parsimony-informative sites being confirmed. The concatenated tree built with the nucleotide sequence clustered the investigated three isolates originating from the three Grey Slender Lorises and shared 100% sequence similarity (with 0.00 genetic distance) and bootstrap supports of 100%. However, the reference strains *T. pyogenes* DSM 20594 and *T. pyogenes* DSM 20630^T^ shared with 98.9% and 99%, respectively the pairwise nucleotide sequence similarity (with 0.012 and 0.010 genetic distance) with the three isolates originating from the three Grey Slender Lorises (Fig. 3a – Table 4). The nucleotide composition (GC content) of the four investigated target genes (*fusA-tuf-metG-gyrA*) of the three *T. pyogenes* from the Grey Slender Lorises, *T. pyogenes* DSM 20594 and *T. pyogenes* DSM 20630^T^ exhibited an identical GC content of 61.3 mol% and 54.7 mol% for the reference strain *A. haemolyticum* DSM 20595^T^. However, the percentage in the content of Guanine and Cytosine were identical for the three *T. pyogenes* isolates originating from the Grey Slender Lorises (29.7 mol%, 31.6 mol% respectively) and for the two reference strains *T. pyogenes* DSM 20594 and *T. pyogenes* DSM 20630^T^ (29.8 mol% and 31.5 mol% respectively) (Table 5). The concatenated sequence of the four loci on the nucleotide level shows clearly on 15 different sites that the three *T. pyogenes* isolates originating from the Grey Slender Lorises were identical and differed from the other three reference strains (Table 6). On the other hand, the concatenated sequence of the four loci on the amino acid level among the 6 investigated isolates and strains revealed 1103 sites with 570 conserved, 217 variables sites and 1 parsimony-informative site. The concatenated tree built with the amino acid sequence succeeded in clustering the three investigated *T*. *pyogenes* isolates originating from the three Grey Slender Lorises and separating them from the other reference strains (*T. pyogenes* DSM 20594, *T. pyogenes* DSM 20630^T^ and *A. haemolyticum* DSM 20595^T^). The three isolates originating from the three Grey Slender Lorises shared 100% amino acid sequence similarity and bootstrap supports of 87%, while the reference strains *T. pyogenes* DSM 20594 and *T. pyogenes* DSM 20630^T^ shared 99.7% amino acid sequence similarity with the three isolates originating from the three Grey Slender Lorises (Fig. 3b). The percentage of amino acid similarities among *T. pyogenes* species and *A. haemolyticum* DSM 20595^T^ ranged from 80.3% to 80.4%, while within the *T. pyogenes* species it ranged from 99.6% to 100%. Moreover, the concatenated sequence of the four loci on the amino acid level clearly showed on the site 248 that the three *T. pyogenes* isolates of Grey Slender Loris origin were identical (Aspartic acid) and differed from the other three reference strains (Glutamic acid).

**Table 3:** GenBank accession numbers of locus sequences of *T. pyogenes* strains obtained in this study

	Isolates and strains	*fusA*	*Tuf*	*metG*	*gyrA*
1	*T. pyogenes* 121,018,522	KJ605914	HG941714	HG941711	HG941706
2	*T. pyogenes* 121,008,157	KJ605913	HG941713	HG941710	HG530074
3	*T. pyogenes* 11-07-D-03394	KJ605912	HG941712	HG941709	HG941702
4	*T. pyogenes* DSM 20630^T^	KJ605911	HG941716	HG941708	HG941704
5	*T. pyogenes* DSM 20594	KJ605910	HG941715	HG941707	HG941703

**Table 4:** Average pairwise distances of the concatenated sequences of the investigated strains

	Isolates or strains	1	2	3	4	5	6
1	*T. pyogenes* 121,018,522						
2	*T. pyogenes* 121,008,157	0					
3	*T. pyogenes* 11-7-D-03394	0	0				
4	*T. pyogenes* DSM 20630^T^	0.010	0.010	0.010			
5	*T. pyogenes* DSM 20594	0.012	0.012	0.012	0.010		
6	*A. haemolyticum* DSM 20595^T^	0.249	0.249	0.249	0.25	0.249

**Table 5:** Nucleotide percentage of the concatenated sequences for the four loci sequences

	Isolates and strains	T	C	A	G	G + C
1	*T. pyogenes* 121,018,522	18.3	31.6	20.5	29.7	61.3
2	*T. pyogenes* 121,008,157	18.3	31.6	20.5	29.7	61.3
3	*T. pyogenes* 11-7-D-03394	18.3	31.6	20.5	29.7	61.3
4	*T. pyogenes* DSM 20630^T^	18.3	31.5	20.5	29.8	61.3
5	*T. pyogenes* DSM 20594	18.3	31.5	20.5	29.8	61.3
6	*A. haemolyticum* DSM 20595^T^	22.9	26.9	22.4	27.8	54.7

**Table 6:** Matrix of the variable nucleotide positions of the concatenated sequences among the investigated strains

Isolates and strains	Nucleotide position																											
				1	1	**1**	**1**	**1**	1	1	1	1	1	**1**	**1**	**1**	**1**	2	2	**2**	2	2	**2**	**3**	**3**	3	**3**	3
	**1** ^**a**^	**4**	**7**	0	2	**2**	**3**	**4**	5	5	8	8	8	**8**	**9**	**9**	**9**	0	1	**5**	7	7	**8**	**0**	**1**	2	**2**	3
	**6**	**3**	**4**	4	4	**6**	**9**	**8**	5	8	1	1	3	**5**	**3**	**4**	**5**	3	9	**5**	3	9	**9**	**7**	**3**	4	**5**	1
	**2**	**5**	**4**	4	2	**3**	**5**	**8**	7	8	5	8	6	**7**	**5**	**7**	**6**	1	9	**6**	3	6	**8**	**8**	**3**	6	**5**	6
(1) *T. pyogenes* 121018522	T	C	T	C	T	A	T	G	C	T	A	A	T	C	C	T	A	C	C	T	C	C	T	A	T	C	T	C
(2) *T. pyogenes* 121008157	.	.	.	.	.	.	.	.	.	.	.	.	.	.	.	.	.	.	.	.	.	.	.	.	.	.	.	.
(3) *T. pyogenes* 11-7-D-03394	.	.	.	.	.	.	.	.	.	.	.	.	.	.	.	.	.	.	.	.	.	.	.	.	.	.	.	.
(4) *T. pyogenes* DSM 20630^T^	C	T	G	.	.	G	C	C	T	C	G	G	C	T	T	A	G	.	.	C	.	T	A	G	C	T	C	.
(5) *T. pyogenes* DSM 20594	C	T	G	T	C	G	C	C	T	C	G	G	C	T	T	A	G	T	T	C	T	.	A	G	C	.	C	T
(6) *A. haemolyticum* DSM 20595^T^	C	A	A	T	C	T	C	C	.	.	.	.	.	G	T	G	G	T	T	C	T	T	C	G	C	T	G	T

^a^The nucleotide positions in the three *T. pyogenes* from Grey Slender Lorises are identical and differ from other reference strains

## Conclusion

The several genetic markers of the presented MLSA approach showed clearly that the three *T. pyogenes* originating from Grey Slender Lorises and the two *T. pyogenes* reference strains belong to three different clonal complexes, respectively. The results of the present investigation which represent the first detailed epidemiological study of *T. pyogenes* of this origin clearly indicated that all three *T*. *pyogenes* which contributed with other potentially pathogenic bacteria to the septicemia of the three lorises, respectively shared a clonal origin. However, whether the cross infection between the three animals with *T. pyogenes* isolates, which was present in the lorises’ terrarium over a certain period of time, occurred because of direct contact or a lack of disinfection of the animal facility after detecting the first or the second case remains unclear.

## Abbreviations

(GTG)_5_: Repetitive bacterial DNA elements
*A*: *Arcanobacterium*
ERIC: Enterobacterial repetitive intergenic consensus
MLSA: Multilocus sequence analysis
nt: Nucleotide
RAPD: Randomly amplified polymorphic DNA
rep-PCRs: Repetitive sequence-based polymerase chain reaction
*T*: *Trueperella*
